# Revisiting the monocrotaline‐treated rat as a model of inflammatory lung disease: COVID‐19 and future pandemic threats?

**DOI:** 10.1002/ame2.70099

**Published:** 2025-10-17

**Authors:** Luke P. Kris, Dani‐Louise Dixon, Shailesh Bihari, Jillian M. Carr

**Affiliations:** ^1^ College of Medicine and Public Health Flinders University Adelaide South Australia Australia; ^2^ Flinders Health and Medical Research Institute Flinders University Adelaide South Australia Australia; ^3^ ICU, Flinders Medical Centre Adelaide South Australia Australia

**Keywords:** COVID‐19, inflammation, monocrotaline, rat model, respiratory

## Abstract

The COVID‐19 pandemic posed a challenge for clinical management of a new lung disease that was characterized by inflammation, endothelial cell dysfunction, and thrombosis, which occur after the replication phase of infection of severe acute respiratory syndrome coronavirus 2 (SARS‐CoV‐2). There are many laboratory models of active SARS‐CoV‐2 infection in mice, reflecting an acute lung injury in an otherwise healthy animal, but there is a lack of accurate animal models of the postviral inflammatory phase of the COVID‐19 lung reflecting severe disease. The monocrotaline (MCT)–treated rat is a widely used laboratory model of pulmonary hypertension (PH). Not often discussed, however, are the observed changes in inflammation, edema, fibrosis, and microthrombosis in the lung prior to PH. At the cellular level, there is loss of pneumocytes and endotheliopathy, and at the molecular level the MCT rat lung is characterized by a pro‐inflammatory cytokine profile, namely elevated interleukin 6, transforming growth factor β and tumor necrosis factor, M1 macrophage phenotype, and dysregulation of the angiotensin converting enzyme (ACE)/ACE2 balance. The systems‐level pathophysiology of the MCT‐treated rat includes progressive cardiopulmonary dysfunction. The MCT‐treated rat clearly differs from the COVID‐19 lung in terms of the triggers for pathology, but there are many parallels apparent in both the MCT‐treated rat and the COVID‐19 lung. The MCT‐treated rat lung as a model of the COVID‐19 lung may provide an in‐depth understanding of the factors that drive the lung to more severe pathology, treatments that benefit lung recovery, or the factors that prove a useful research platform for future emerging respiratory threats of similar pathology.

## Graphical abstract

In this review, the pathological, molecular, and cellular changes observed in the COVID‐19 lung are compared to those in the laboratory rat treated with monocrotaline (MCT). Similarities that reflect common changes associated with inflammation and endothelial cell dysfunction are observed in both states, leading to a lung pathology characterized by edema, thrombosis, and fibrosis. Could this model be used to understand the postviral inflammatory phase of the COVID‐19 lung, the triggers that worsen COVID‐19 pathology, or the ways that could benefit in lung repair and recovery?

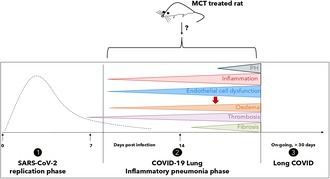

## 1 INTRODUCTION

**FIGURE 1:**
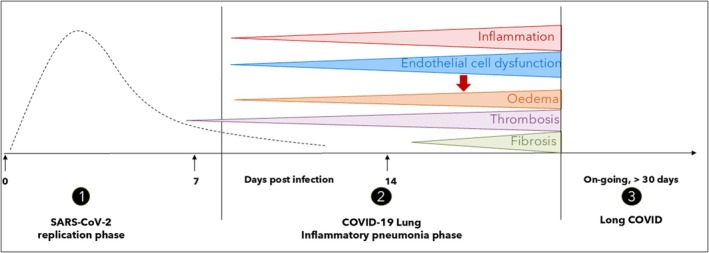
The phases of SARS‐CoV‐2 (severe acute respiratory syndrome coronavirus 2) and COVID‐19. Schematic representation of the timing of (1) the acute phase of SARS‐CoV‐2 infection and viral replication, occurring approximately within the first 7–10 days; (2) the postviral inflammatory phase that includes the COVID‐19 lung and pneumonia; and (3) the ongoing sequalae after SARS‐CoV‐2 infection, typically cited as >30 days of persistent symptoms and termed “long COVID” or PASC (postacute sequelae of COVID‐19).

Rats have been used extensively to study acute respiratory distress syndrome (ARDS) using models such as lipopolysaccharide (LPS) stimulation or chemical treatment such as with bleomycin or oleic acid.^1^, ^2^, ^3^ These treatments induce acute damage to the respiratory epithelium and cause alveolar inflammation and endothelial cell (EC) dysfunction, reflecting the major pathologies of the diverse ARDS presentation in humans.^4^ Severe acute respiratory syndrome coronavirus 2 (SARS‐CoV‐2) infection and the COVID‐19 lung now present another trigger of ARDS and inflammatory lung disease. Laboratory animal models for COVID‐19 have been developed substantially since the start of the pandemic, where initially SARS‐CoV‐2 did not infect laboratory mice. Although now there are many laboratory animal models to study acute SARS‐CoV‐2 infection and replication,^5^, ^6^, ^7^ and a recognition for a need of animal models of the sequalae of COVID‐19 such as “long COVID” or postacute sequelae of COVID‐19,^8^ there is a lack of models to reflect the later postviral inflammatory lung pathology (Figure 1). Additionally, many animal models are based on nonphysiological overexpression of human angiotensin converting enzyme 2 (ACE2), a key molecule in the balance of the renin–angiotensin system (RAS) and known to influence inflammatory and thrombotic lung disease.^9^, ^10^, ^11^, ^12^, ^13^ SARS‐CoV‐2 infection of the Syrian golden hamster, nonhuman primates such as the rhesus macaque, or mouse‐adapted SARS‐CoV‐2 are more accurate models of viral‐induced pneumonia and the COVID‐19 lung, which can also reflect the impact of age.^14^, ^15^ Models of the Syrian golden hamster or rhesus macaque are expensive and not widely available.^16^ The rat is a readily available laboratory animal, is easy to house and maintain, and is manipulatable in terms of laboratory measurements of respiratory function, particularly in comparison to the difficulties of these measurements in mice.^17^ Here, the physiological, cellular, and molecular characteristics of an alternative laboratory model of lung injury, the monocrotaline (MCT)‐treated rat, are described and contrasted to the changes observed in the COVID‐19 lung. Our aim was to assess the alignment of the MCT‐treated rat lung with the postviral inflammatory phase of COVID‐19 lung disease, with the future view as a research model where stimuli or treatments in the laboratory setting can be tested for the impact of worsening or improving the pathology of the lung. Additionally, such a model would be noninfectious and benefit from avoiding specialized biocontainment needs in the laboratory.

## 2 THE PHYSIOLOGY OF THE COVID‐19 LUNG

For quite some time, the predominant hospital presentation of COVID‐19 was respiratory distress and dyspnea, with pulmonary damage leading to respiratory failure.^18^ Today, with vaccination and infection‐induced immunity prevalent, the impact of COVID‐19 is declining. However, data from the World Health Organization, March 12, 2025, report collating COVID‐19 data from 103 countries over a 4‐week period, January 6 to February 2, demonstrate that there are still over 16 700 new hospitalizations, 700 new intensive care unit admissions, and 4500 fatalities from 147 000 reported cases.^19^ Thus, it is clear that COVID‐19 can still be a severe disease. It is contentious whether this presentation is definitively classifiable as severe pneumonia, ARDS, or a distinct manifestation that shares characteristics of both pathologies, and the term “COVID‐19 related ARDS” has been applied. Secondary bacterial or fungal infections are recognized in COVID‐19 patients and contribute to increased mortality.^20^, ^21^ This review focused on the lung pathology of primary postviral COVID‐19‐related illness (Figure 1), for assessing the applicability of this model for testing stimuli, such as bacterial or fungal infection, that worsen COVID‐19 disease.

Early in the pandemic, computed tomography scans of the COVID‐19 lung at the time of patient presentation to hospital commonly identified bilateral, multifocal, and peripherally distributed ground glass opacities (GGO).^22^, ^23^ GGOs can become more confluent, developing into subpleural frank consolidation, with reticular patterns also emerging, indicative of bronchiolectasis and interlobular septal thickening.^22^ Such findings may also be superimposed with consolidation, forming a “crazy paving” pattern.^24^ Morphologically, the COVID‐19 lung observed during the pandemic changed along a dynamic temporal continuum. In a four‐phase dogma put forth by Bosmuller et al.,^25^ the early stage involved edema, with epithelial damage and endothelitis/capillaritis.^26^ Diffuse alveolar damage (DAD) then ensued, with the exudative stage within the first week; organizing stage, one to several weeks; and fibrotic stages, weeks to months postinfection. This highlights the extensive time frame of changes in the COVID‐19 lung relative to the acute viral phase of infection (Figure 1). Additionally, not uncommon was pulmonary vascular enlargement. For example, in a cohort of 87 COVID‐19 patients, vascular enlargement was observed in 75.9%, including 100% of severe or critically ill patients.^27^ This pulmonary vasculopathy and vascular thickening also reflects the key features of pulmonary hypertension (PH).^28^ Additionally, right ventricle (RV) dilation and/or failure is reported in COVID‐19 patients with acute pulmonary injury.^29^, ^30^ It has since been well established that COVID‐19 is associated with acute and chronic cardiovascular dysfunction, as reviewed.^31^, ^32^ For instance, COVID‐19 is worse in patients with preexisting cardiovascular disease, although the infection itself can induce myocarditis and myocardial injury^33^ with ongoing risks, such as increased cardiovascular events up to 1‐year post‐COVID‐19^34^ and elevated incidence of thrombotic or vascular events up to 49 weeks post‐COVID‐19.^35^ Therefore, any COVID‐19 model should also consider the cardiac and vascular impacts of infection.

Postmortem analyses of the COVID‐19 lung corroborate the clinical findings, and a meta‐analysis has been presented.^26^ For example, postmortem analysis of 32 fatal COVID‐19 cases found that most patients (75%) exhibited signs of both exudative and proliferative DAD.^36^ Epithelial damage, exudate formation, and vascular pathology are the predominant histopathological findings at autopsy.^25^, ^26^ Particularly, pneumocyte denudation, type 2 pneumocyte hyperplasia, fibrin, and macrophage‐rich edematous infiltrate are observed.^25^, ^26^ Microvascular thromboses are also well described and have been identified as a major feature of the COVID‐19 lung. Consistent with this, gross postmortem examination often reveals increased lung weight due to diffuse parenchymal edema and congestion, hemorrhagic necrosis, infarction, and/or pulmonary thrombi.^37^ The diversity of lung pathology at autopsy is also observed.^38^

At a functional level, two different phenotypes of the COVID‐19 lung were initially described early in the pandemic: the “H” and “L” phenotypes.^39^, ^40^ In a subset of hospitalized COVID‐19 patients, lung compliance and diffusive capacity were diminished, with an increase in right‐to‐left shunting and an accordingly high V/Q ratio.^26^, ^39^ SaO_2_ can decrease to less than 94% on room air, and PaO_2_/FiO_2_ ratios often meet the Berlin definition's criterion for ARDS of ≤300 mmHg. For instance, in a 2020 study of 1028 patients across Italy, median PaO_2_/FiO_2_ at admission was 196.43 mmHg.^41^ This “H” (high‐elastance) lung phenotype is associated with increased recruitability and therapeutic response to positive end‐expiratory pressure ventilation. In the juxtaposing “L” (low‐elastance) phenotype, lungs have high compliance with normal tidal volumes and low recruitability. Despite preserved lung mechanics, L‐phenotype patients present with moderate to severe hypoxemia. Such hypoxemia presents without dyspnea and is termed “happy hypoxemia,” which made clinical management difficult in that ventilation of the poorly recruitable lung, with the intention of stabilizing PaO_2_, can paradoxically lead to ventilator‐induced lung injury.^42^ It has been suggested, however, that rather than separate pathophysiologies, L and H phenotypes represent stages of evolving disease,^43^, ^44^ and in the current era, severe COVID‐19 less often follows the dichotomy of an L or H phenotype. However, an improved understanding of the relationship between the clinical lung phenotype with noninjurious oxygen management is important,^43^, ^44^ and improved laboratory models of these lung phenotypes would help inform better clinical management strategies for oxygenation and ultimately recovery of the COVID‐19 lung or similar new emerging pathologies.

## 3 CELLULAR AND MOLECULAR CHANGES IN THE COVID‐19 LUNG

The key cell types for SARS‐CoV‐2 infection and COVID‐19 lung pathology include respiratory epithelial cells,^45^ ECs, and alveolar macrophages. An RNAseq “atlas” of tissues from COVID‐19 autopsies identified altered gene expression in epithelial cells and alveolar type II cells, and viral RNAs in mononuclear cells and ECs of the lung.^46^ Further RNAseq analysis characterized lung inflammation, including interleukin 1β (IL‐1β) and IL‐6 induction, activation of alveolar macrophages, and changes in alveolar type II cells associated with reduced ability to generate reparative alveolar type I cells.^47^ These findings substantiate a cellular respiratory epithelial cell insult, followed by infection or activation of other cell types, such as the endothelium and alveolar macrophages in the COVID‐19 lung. Further, functional changes in alveolar macrophages are associated with COVID‐19 disease, with an increase in inflammatory monocyte/macrophages associated with more severe disease^48^ and the presence of infected alveolar macrophages at autopsy.^49^ Profibrotic gene expression profiles from macrophages in the COVID‐19 lung at autopsy are observed, with these macrophage expression profiles replicated in monocytes exposed to SARS‐CoV‐2 in vitro.^50^ Von Willebrand factor (VWF), soluble P‐selectin, and D‐dimers are increased in COVID‐19 patients with more severe pulmonary disease (as measured by oxygen requirement), and these markers of coagulation correlate with markers such as monocyte chemotactic protein (MCP‐1)/C‐C motif ligand 2 (CCL2), tumor necrosis factor (TNF), CXCL10, and vascular cell adhesion molecule 1 and suggest both macrophage inflammatory response and EC activation.^51^ These combined cellular effects of the virus on the alveoli and the vasculature, including inflammation and thrombosis, are proposed to underlie the subsequent poor oxygenation observed in COVID‐19 patients.^52^

At the molecular level, SARS‐CoV‐2 infection leads to the activation of pathogen recognition pathways such as toll‐like receptor (TLR) 2^53^ and likely TLR3, with subsequent induction of type I interferon (IFN) and IFN‐stimulated antiviral genes.^54^, ^55^ SARS‐CoV‐2 also induces innate inflammatory responses, such as NFkβ (nuclear factor kappa beta) and NLRP3 (NOD‐like receptor family pyrin domain containing 3) inflammasome activation,^56^ leading to a pro‐inflammatory “cytokine storm”,^57^ including increased production of TNF, IL‐6, IFN‐ϒ, and IL‐1β.^54^, ^58^, ^59^ The complement system is also activated^60^ and transforming growth factor (TGF) β is increased, with reported abhorrent TGF‐β signaling.^61^ Both these molecular systems are likely key to vascular dysfunction, based on well‐described roles such as complement product in‐mediated EC permeability and pulmonary injury^62^ and altered TGF‐β/bone morphogenic protein‐2 (BMP2) receptor‐mediated induction of factors such as vascular endothelial cell growth factor (VEGF). The cells contributing to this cytokine storm and pro‐inflammatory milieu in this late phase of disease are likely alveolar and infiltrating macrophages,^63^ although neutrophils may also play a role.^64^

In addition to these innate responses, SARS‐CoV‐2 downregulates ACE2 on the cell surface, which likely contributes to the viral‐induced inflammatory response.^65^, ^66^ ACE and ACE2 are cell membrane–bound and fluid‐phase enzymes that regulate RAS, a key regulator of blood pressure. ACE cleaves angiotensin I (AngI) into AngII, which acts via the AngI receptor (AT1R) to induce inflammation, fibrosis, and EC dysfunction. Subsequently, AngII is cleaved by ACE2 into Ang1‐7, which stimulates anti‐inflammatory and antifibrotic responses and vasodilation.^67^ Thus, the AngI/AngII/Ang1‐7 balance, and therefore the ACE/ACE2 ratio, is important in controlling damaging versus reparative processes in the lung, as is exemplified by the association of ARDS with genetic polymorphisms that lead to high levels of ACE.^68^, ^69^ Therefore, whereas the RAS is key to blood pressure control, the relative ratio of ACE/ACE2 is otherwise important, where high ACE/AngII drives pro‐inflammatory responses in the lung, high ACE2 reduces the pathology of ARDS and reciprocally, low ACE2 is likely an important contributor to inflammation in the COVID‐19 lung.^9^, ^10^, ^11^, ^12^, ^13^ In addition to inflammation, induction of EC permeability and changes in coagulation contribute to major COVID‐19 issues of edema and thrombosis. These effects can be mediated by direct viral damage of the endothelium, reducing the production of factors such as prostaglandin E2 and inducing the release of procoagulant peptides from intracellular stores, including factor VIII and VWF,^25^, ^26^, ^70^ or as described earlier through vasoactive factors such as complement components, VEGF or TGF‐β. Additionally, the pro‐inflammatory milieu described earlier promotes coagulation through EC production of factors such as thromboxane, antiplasmin, and plasminogen activator inhibitor 1 (PAI‐1).

Thus, the changes in the COVID‐19 patient lung are well documented. In an aged mouse model of mouse‐adapted SARS‐CoV‐2 challenge, the EC dysfunction and thrombotic effects of the COVID‐19 lung are replicated, with similar molecular changes in gene expression, such as in PAI‐1and P‐selectin.^15^, ^51^ Interestingly, recent data from this model have suggested that EC senescence in the aged mouse lung is associated with SARS‐CoV‐2‐induced vascular dysfunction and a more severe lung phenotype.^71^ A recent comparative transcriptional analysis of nasopharyngeal aspirates (NPA) taken during acute SARS‐CoV‐2 infection and the COVID‐19 lung at autopsy has highlighted progression from a viral inflammatory phase and acute responses to the later postviral COVID‐19 lung.^72^ The transcriptional profile of the NPA demonstrated an acute antiviral response, with high levels of viral RNA, and increased canonical innate IFNs stimulated genes and double‐stranded RNA responses such as interferon‐induced protein with tetratricopeptide repeats (IFIT) 2; 2'‐5' oligoadenylate synthetase (OAS) 1, OAS2, and OAS3; and interferon stimulated gene (ISG) 15.^72^ In contrast, in the COVID‐19 lung at autopsy there were no detectable viral RNA transcripts but ongoing inflammation with increased factors such as TNF and IL‐6, increased Th1 cytokines typically associated with pro‐inflammatory chemotaxis such as CXCL10 and CCL2, and innate antiviral responses such as MX1.^72^ Additionally, there was activation of the RAS with downregulation of ACE2 and angiotensin receptor 2, which would be associated with promoting inflammation. Although the complement classical pathway regulator SERPING1 (also known as C1 inhibitor, C1‐INH, with serine protease activity) is upregulated in NPA, SERPING1 is downregulated in the COVID‐19 lung, suggesting excess activity of the classical complement pathway and potential additional impact on clotting propensity in the COVID‐19 lung.^72^ Further impact of fibrin and hyaluronic acid deposition in the COVID‐19 lung is suggested by increased transcripts for genes promoting fibrin synthesis, hyaluron synthase isoenzymes, and SERPINE1, an inhibitor of fibrinolysis.^72^ Transcriptional changes also suggest mitochondrial dysfunction, with hypoxia characterized by increased HIF‐1a in the lung. Many of these transcriptional changes in the COVID‐19 lung were also reflected in the Syrian golden hamster or mouse models of post‐SARS‐CoV‐2 pneumonia.^72^ However, in the COVID‐19 autopsy samples, inflammatory transcriptional profiles, in the absence of viral transcripts, were also observed in organs such as the kidney and heart.^72^ This inflammatory state in organs other than the lung was also observed in the Syrian golden hamster but not in the mouse models of post‐SARS‐CoV‐2 pneumonia. Overall, this recent transcriptional profiling strengthens our understanding of COVID‐19 as an inflammatory, fibrotic, thrombotic state, with an RAS imbalance, in the absence of viral replication, and with accompanying multiorgan dysfunction, that is reflected by postviral states in the Syrian golden hamster but not in more common laboratory mouse models. These parallels in the Syrian golden hamster and the aged mouse‐adapted mouse challenge models to the COVID‐19 lung are encouraging, but a model in the absence of infectious virus, in a more manipulable and readily available animal such as the rat, would also be of benefit.

A summary of the clinical and autopsy findings and cellular and molecular changes in the COVID‐19 lung is presented in Table 1.

**TABLE 1:** Comparison of the major pathologies of the COVID‐19 lung with the low‐dose, early MCT‐treated rat.

	Low‐dose, MCT‐treated rat	Human COVID‐19 lung
**Time frame for disease**	7–14 days post‐MCT	After the decline in viremia, ~7–10 days
**Lung tissue pathology**		
Alveolar damage	Yes	Yes, diffuse
Edema	Yes	Yes
Fibrosis	Yes, later (21–28 days)	Yes
Cellular infiltrate	Yes, neutrophils and M1 macrophage	Yes, neutrophils and M1 macrophage
Microvascular thrombi	Yes	Yes
**Cardiopulmonary**		
Pulmonary artery thickening	Yes, initiating/progressing with time	Yes
Cardiac failure/damage	Yes, later (20–40 days) RV, and direct cardiotoxicity	Yes, predominantly RV
Myocarditis	Yes, isolated LV (late, ~40 days)	Yes, including post‐COVID‐19 recovery
Pulmonary hypertension	Yes, progressive, significant from ~21 days	Yes
**Cellular and molecular changes in the lung**		
Damage/death of respiratory epithelium	Yes	Yes
ATII cell dysfunction	Yes	Yes
Activation of macrophage M1 pro‐inflammatory state	Yes	Yes
Endothelial cell damage and activation	Yes	Yes
Increased endothelial cell permeability	Yes	Yes
Renin–angiotensin system	Increase in ACE and AGTR1, decrease in ACE2	Decreased ACE2 and AGTR2
Inflammatory changes	Pro‐inflammatory	Pro‐inflammatory^a^
CCL2	Induced	Induced
TNF	Induced	Induced
IL‐6	Induced	Induced^a^
IL‐1β	Induced	Induced
TGF‐β	Induced, dysfunctional TGF‐β signaling by MCTP binding to the BMP2 receptor	Induced, dysfunctional TGF‐β signaling
NLRP3 inflammasome	Induced	Induced
Coagulation and thrombosis	Increased, multiple mechanistic changes	Increased, multiple mechanistic changes^a^

*Note*: See text for specific supporting references and detail.

Abbreviations: ACE, angiotensin converting enzyme; AGTR, angiotensin II receptor; BMP, bone morphogenic protein; CCL2, C‐C motif ligand 2; IL, interleukin; LV, left ventricle; MCT, monocrotaline; MCTP, monocrotaline pyrrole; NLRP3, NOD‐like receptor family pyrin domain containing 3; RV, right ventricle; TGF, transforming growth factor; TNF, tumor necrosis factor.

^a^ Targeted therapeutically.

## 4 IS THE MCT RAT LUNG REFLECTIVE OF COVID‐19 LUNG PATHOLOGY?

MCT is a pyrrolizidine alkaloid toxin that is metabolized primarily by the liver, forming the active agent dehydromonocrotaline or monocrotaline pyrrole (MCTP). The main use of the MCT‐treated rat model is in the study of PH, with progressive arterial thickening and fibrosis of the large vessels of the lung.^73^ For example, 60–80 mg/kg of MCT administered via the intraperitoneal or subcutaneous route to adult Sprague–Dawley rats results in PH at 21–28 days post‐MCT, which can progress to RV cardiac failure as reviewed.^74^, ^75^ Additionally, independent of PH, MCT can have a direct impact on the heart with RV myocardium hypertrophy^76^ and left ventricle (LV) atrophy and myocardium deterioration with a cellular infiltrate indicative of LV myocarditis.^77^

At earlier time points in the MCT rat model however, before pathological PH, the disease reflects lung inflammation with dysfunction of EC and hypercoagulability. Airway function is reduced with a deterioration in pulmonary mechanics and gas exchange^78^ and a decrease in diffusing capacity of the lungs for carbon monoxide (DLCO) prior to major pulmonary vascular abnormalities.^74^, ^79^ A PaO_2_/FIO_2_ value of <300 mmHg (reflective of the Berlin definition of ARDS in humans), interstitial and alveolar granulocyte infiltration, and microvascular leakage leading to edema are also reported in MCT‐treated rats at early time points.^74^ Whereas RV insufficiency is apparent at 28 days post‐MCT, at 14 days the pathology is milder and reflective of increased pulmonary vascular resistance.^80^ Analysis of the time course of MCT‐induced inflammation in the lung demonstrates an induction of pro‐inflammatory factors, TNF and IL‐1β, and an M1 macrophage phenotype at 6–9 days post‐MCT treatment.^81^ RNAseq of the response to MCT has shown that as early as 1–2 weeks there is differential expression of genes in the lung reflecting induction of innate immune responses and cytokine‐chemokine pathways,^82^ including TLR pathways and activation of the NLRP3 inflammasome. Additionally, NFkB transcriptional pathways are activated, and IL‐6 is induced.^82^ MCT (or the active metabolite MCT‐P) also induces thrombi in the microvasculature of the lung,^83^ with increased circulating fibronectin,^84^ increased platelet sequestration,^85^ and increased tissue factor expression,^86^ suggestive of hemostatic dysfunction. Upregulation of both the coagulation cascade and complement pathways is evident by proteomic analysis of lung tissue from MCT‐treated rats, as early as 1–2 weeks posttreatment.^87^

At the cellular level MCTP acts by binding to the extracellular calcium sensing receptor and the BMP2 receptor on EC, leading to dysregulated BMP2 signaling.^88^ The ligand for BMP2 is TGF‐β,^89^ and introduction of BMP2‐transduced endothelial progenitor cells into a model of LPS‐induced ARDS in rats improves lung function, highlighting the central roles of these signaling pathways, and EC, in the pathogenesis of ARDS.^90^ Additionally, as in COVID‐19, RAS is disrupted in the MCT rat,^91^ although in the MCT rat, these changes are affected at the messenger RNA (mRNA) level and temporally. Further, at later time points post‐MCT treatment, the changes in the RAS are driven by the kidney,^92^ rather than the lung as in COVID‐19. For instance, high ACE and AT1R mRNA levels are observed in the MCT rat lung^91^ in association with increased inflammatory cytokines TGF‐β, TNF, IL‐6, IL‐1, and MCP‐1/CCL2 and decreased anti‐inflammatory IL‐10.^91^ Additionally, MCT induction of PH in combination with hypoxia results in reduced ACE2 mRNA and protein in the lung.^93^ In contrast, at later times (28 days) after MCT treatment of rats, elevated ACE mRNA is observed in the kidney and heart, whereas ACE mRNA is reduced in the lung, with increased renin production from the kidney.^92^ Macrophages and EC responses likely coordinate to produce a damaging lung microenvironment after MCT treatment, where MCT treatment activates the NLRP3 inflammasome in monocyte/macrophages that subsequently induce ferroptosis in EC of the lung.^94^ Thus, the low‐dose, early post‐MCT‐treatment rat reflects an inflammatory lung disease with endotheliopathy, coagulopathy, and a developing PH. This model aligns well with the COVID‐19 lung in terms of (i) overall lung pathology of edema, fibrosis, and microthrombi; (ii) the cell types involved with damage to the respiratory epithelium and endothelium, and activation of alveolar macrophages; (iii) induction of key pro‐inflammatory cytokines and RAS; and (iv) associated impact on the heart. This is contrasted in Table 1.

## 5 LIMITATIONS

Although Table 1 contrasts many similarities, every model has its limitations. In the MCT‐treated rat, the mechanisms driving the lung dysfunction are clearly different from those of the COVID‐19 lung. For instance, the initial trigger for downregulation of ACE2 in COVID‐19 is through SARS‐CoV‐2 binding, whereas in the MCT‐treated rat, as described earlier, this is believed to be mediated by mRNA changes and further influenced by changes in the RAS induced through MCT impact on hypoxia and the kidney.^91^, ^92^, ^93^ Although the transcriptomic data in humans and hamster models of COVID‐19^72^ also clearly show inflammatory responses in the kidney and heart, the major COVID‐19 pathology remains an inflammatory lung disease; in contrast, the major pathology of the MCT‐treated rat is ultimately a PH, secondary to an inflammatory lung state. Further, parallels are clear in the comparable cytokines and chemokines that are altered in the COVID‐19 and the MCT‐treated rat lung (Table 1), but the degree of these changes and therefore the balance of the pro‐inflammatory state may differ between the COVID‐19 and MCT rat lung and have not been directly compared. Additionally, although it is possible to include other influences of COVID‐19 disease, such as age and obesity, in the MCT‐treated rat model, the model is still of restricted genetic diversity compared to humans, where sequence variations in ACE2,^95^ expression of TLR3 or TLR4,^96^ or preexisting type I IFN autoantibodies^97^ can impact COVID‐19 severity in humans. Finally, although the rat is a widely used model for human respiratory disease, there are known differences in the tracheobronchial branching patterns and lobar structures of the rat lung compared to humans.^98^, ^99^

## 6 CONCLUSIONS AND FUTURE PERSPECTIVES

Pathogen‐driven lung disease is a major global burden with ever‐changing and emerging viral threats. SARS‐CoV‐2 infection is a recent and significant example, where the early viral phase of illness reflects an antiviral and inflammatory response that usually resolves, and the virus is cleared. Sometimes however, a postviral inflammatory phase ensues, and this is the phase of disease that can lead to severe, life‐threatening illness, as is seen with COVID‐19.^100^, ^101^, ^102^ In 2025, the risk of severe COVID‐19 is reduced due to widespread population vaccination in most high‐ and upper‐middle‐income countries^103^ and the availability of successful SARS‐CoV‐2 antiviral therapy.^104^, ^105^ SARS‐CoV‐2 variants, however, have emerged and will continue to be produced with molecular changes that can influence neutralizing antibody binding and viral entry mechanisms, as reviewed.^106^ SARS‐CoV‐2 infection and COVID‐19 severe respiratory illness have now become a permanent addition to the regular human respiratory challenges. Laboratory animal models are therefore necessary to study COVID‐19 pathophysiology, as they may ultimately facilitate the development of management and treatment strategies. The literature summarized earlier suggests a benefit for revisiting the MCT rat as a simple, flexible, and adaptable laboratory model of inflammatory, fibrotic, and thrombotic lung disease associated with epithelial damage, alveolar macrophage inflammation, and RAS and EC dysfunction. In addition to COVID‐19, a modified MCT rat model could be an easy and defined starting point for laboratory research to rapidly apply to the inevitable future respiratory pandemic threats. Rather than a model of the mechanisms that lead to development of the COVID‐19 lung or ARDS initially, we envisage that the addition of stimuli to the lung of the MCT‐treated rat may be a useful laboratory approach to investigate factors that subsequently exacerbate the COVID‐19 lung pathology, such as secondary bacterial or fungal infection,^20^, ^21^, ^107^ or alternatively treatment or management approaches to help oxygenate, repair, and aid recovery of the COVID‐19 lung.

## AUTHOR CONTRIBUTIONS

**Luke P. Kris:** Conceptualization; data curation; investigation; writing – original draft; writing – review and editing. **Dani‐Louise Dixon:** Conceptualization; writing – review and editing. **Shailesh Bihari:** Conceptualization; writing – review and editing. **Jillian M. Carr:** Conceptualization; data curation; investigation; supervision; writing – original draft; writing – review and editing.

## FUNDING INFORMATION

Luke P. Kris is supported by the College of Medicine and Public Health MD, Advanced Studies Program. Funding was provided by the Australian National Health and Medical Research Council (NHMRC), Ideas Grant GNT2003683.

## CONFLICT OF INTEREST STATEMENT

The authors have no competing interests to declare that are relevant to the content of this article.

## ETHICS STATEMENT

This article is a narrative review of the literature and has no animal or human ethics approvals to declare.
